# Negotiating Ethics-in-Action in a Long-term Research Relationship with a Young Child

**DOI:** 10.1007/s42087-021-00216-z

**Published:** 2021-04-19

**Authors:** Niina Rutanen, Raija Raittila, Kaisa Harju, Yaiza Lucas Revilla, Maritta Hännikäinen

**Affiliations:** Faculty of Education and Psychology, Department of Education, University of Jyväskylä, Jyväskylä, Finland

**Keywords:** Relational ethics, Research space, Early childhood education and care, Transitions, Qualitative case study

## Abstract

This article continues the discussions of relational ethics put forward in *Human Arenas* in “Arena of Ethics” (Hilppö et al., 2019). Our aim in this article is to explore and discuss relational ethics, as ethics-in-action, in a long-term research relationship with a child. Our question is: How is ethics-in-action negotiated during critical incidents in the construction of a research space that involves a long-term research relationship with a young child? This article is based on a research project that focused on children’s transitions in early childhood education and care (ECEC). These transitions include the transition from home care to ECEC as well as transitions from child groups or settings to other ECEC groups or settings, and the transition to pre-primary education. We apply a particular lens to the corpus of data, analyzing and reflecting critical incidents vis-à-vis a negotiation of ethics-in-action during the construction of our research space, which involved a long-term research relationship with a child. Our results show that critical incidents in our study’s negotiation of ethics-in-action included (a) the focus child’s spontaneous contributions to the study’s interviews, (b) interdependencies between the child and diverse researchers, and (c) the child’s evolving expertise in data collection, which restructured our study’s research space. We conclude that ethical questions cannot be separated from the mutually constituted relationships or socio-spatial context in where they emerge; thus, they are relationally and spatially embedded.

## Introduction

This article continues discussions started in *Human Arenas*’ special section on relational ethics for psychological research, “Arena of Ethics” (Freeman, 2019; Chimirri, 2019; Hilppö et al., 2019; O’Doherty & Burgess, 2019; Søndergaard, 2019). These articles powerfully challenge research ethics’ traditional subjectivist theorizing and invite alternative approaches, such as post-humanist theorizing (e.g., Chimirri, 2019) and new materialist thinking (e.g., Søndergaard, 2019), to discuss relational ethics in research involving humans. We use the term *relational ethics* in dialog with, and to complement, the formal ethical procedures that institutional ethics committees have linked to—for example—pre-evaluation processes. *Relational ethics* refers to actual ethical situations that researchers encounter while conducting fieldwork (Ellis, 2007). Relational ethics, in this article, include ideas on (a) the dynamics, unpredictability, and messiness of ethical questions; (b) moral interdependency between researchers and research participants; and (c) understanding that ethical decisions are always socially and spatially situated in a context (Tutenel et al., 2019). In relational ethics, as summarized by Hilppö et al. (2019, 408), “primacy is placed on the relationship between persons and on their constitutive interdependency as the ground for ethical decision-making.” Thus, relational ethical thinking provides space for new and even surprising ethical issues that arise in current research situations. It can, therefore, be understood as *ethics-in-action*.

Our approach to relational ethics is linked to our theoretical approach to studying early childhood education and care (ECEC) based on relational ontology and the concept of *space* (Fuller & Löw, 2017; Lefebvre, 1991; Massey, 2005; Soja, 1996). We conceptualize the research process as a *socially constructed research space* that includes particular, yet continuously evolving, research relationships, and relational ethics—ethics-in-action as a process.

The ethical aspects of research involving young children have been discussed widely in multidisciplinary childhood studies regarding children’s positions and roles in research (Christensen & Prout, 2002; Clark, 2005, 2014; Einarsdóttir, 2007; Hill, 2005; see also Chimirri, 2019). Children have been considered “subjects” and “collaborators” (Grover, 2004) or “participants” in research (e.g., Robson, 2011). Despite broad discussions about children’s positions, roles, power imbalances, and rights as research participants, very young children (e.g., zero-to-two-year-olds, “infants and toddlers”) remain marginalized in these discussions. The youngest children’s nonverbal participation remains somewhat challenging to conceptualize and theorize (White, 2011, 2017b). The few works that have addressed infants’ voices in research have suggested that “hearing children’s voice” in research might, rather than *hearing*, be an act of “seeing”—a co-construction and engagement with children based on ontological assumptions of human dialogicality and relationality (Rayna & Garnier, 2018; White, 2017a, b; see also Freeman, 2019; Hilppö et al., 2019). Relational research ethics face a knowledge gap and a lack of focused studies involving infants and toddlers, as well as a lack of discussions about long-term research relationships that evolve over years with young children.

This lack of discussion is pressing if we follow the conceptualizations that emphasize the constitutive interdependency between persons as the starting point for ethical decision-making (Hilppö et al., 2019). Research process, relationships, and persons are all interdependent and in the process of constitution during the years of the study. Thus, the ethical questions emerge during the research process as a result of these relations that are unique to the study and evolving during the years. To address the ethical questions, it is important to focus on these changing and evolving aspects of the relations and relationships. In addition, with a young child, infant and toddler, it is important to underline their nonverbal contributions that may be easily left unnoticed. It is only through, and in light of, the unique research relationship that a child’s assent for study can be evaluated and interpreted, as will be discussed in this article.

This article scrutinizes one particular case—namely, a research relationship with one child that has lasted approximately five years, as of the time of writing this article. Historically, this type of research relationship has been situated in the years of actively examining and updating agreements for personal data processing with research participants (General Data Protection Regulation [EU] 2016/679).^1^ Parallel to these requirements of scrutinizing personal data processing, various questions on ethics’ processual nature (Hilppö et al., 2019) have been examined. A preverbal one-year-old at the beginning of the research, the focus child turned five years old, began to verbally express opinions, and moved very differently than at the beginning of the process, just to name a few of the changes.

Through this case analysis, our main aim is to explore and discuss relational ethics, as ethics-in-action, focusing on a long-term research relationship with a young child. Our question is: How is ethics-in-action negotiated during critical incidents in the construction of a research space that involves a long-term research relationship with a young child? Methodologically, we analyze and reflect on these “critical incidents” (Halquist & Musanti, 2010; Tripp, 1993) as turning points during the research process.

## Ethical Questions in Research with Children

Ethical questions in research with children are broad. A very simplified characterization is that they range from the informed consent of parents or guardians to children’s own assent to research, which bears some resemblance to discussions of relational ethics (see Hilppö et al., 2019). The informed consent process is considered central in safeguarding children’s rights as research participants (Lambert & Glacken, 2011; Mayne et al., 2016). Researchers have also witnessed increasing requirements from research funders and scientific journals for institutional ethics committees to pre-evaluate research proposals. Thus, much focus and many resources have been allocated to risk management and the protection of children’s rights—even before actual data collection with children begins. Researchers face a challenge to verbalize the actual research process in advance during consent processes and pre-evaluations. However, this detailed planning is not easily negotiated with certain approaches, such as ethnographic methods and long-term engagement with subjects, which always involve unpredictability (see also Tutenel et al., 2019). With older children, who are given a possibility to express written consent, some formal procedures may also pose challenges in reciprocity and mutual understanding (see examples from Kaukko et al., 2019; Ruiz-Casares & Thompson, 2016).

Research with children must follow an ethical commitment to ensuring research subjects’ rights—including rights to protection, privacy, and integrity—and also consider children’s vulnerability. Thus, even if guardians consent to a study, they consent to an initial rough plan that takes shape later in the research process, building on lived ethical experiences. Accordingly, children’s assent to a study could and should be seen as an evolving and dialogical process of shaping the research, as well as the boundaries of both data collection and production (Olli, 2019; see also Einarsdóttir, 2007). For example, Kaukko et al. (2019) argue for understanding the research field with all its relationality, and they emphasize the importance of “hanging-out” in research contexts.

## Relational Ontology and Research Space

This article is based on project Trace in ECEC - Tracing children’s socio-spatial relations and lived experiences in early childhood education transitions (University of Jyväskylä, Finland). The project’s ontological starting point is relational thinking. In short, relational thinking assumes that the world manifests itself to people according to what they are (like) and how they settle themselves in relation to other humans and non-humans in the world. This view attempts to overcome the structure–agency dualism by conceptualizing interdependent relations as the “basic stuff” of social processes (Prandini, 2015). As Donati (2015) states, society is made *by* individuals (not *of* individuals) in relations, and society can be activated only by individuals. Both individuals and the formations they participate in—such as institutions and social systems—belong to the same order of reality, which is a relational order (Prandini, 2015).

This kind of ontology directs our epistemological and methodological thinking in Trace in ECEC - project and steers us to choosing relations—and actions in relations—as research objects. All subjects are involved in the world as participants who affect the rest of the world, and the world also affects subjects. According to Donati (2013, p. 213), the emergence phenomenon (e.g., interview situations) combines elements that had been separate into a new entity. This entity presents properties and powers that cannot be reduced to the sum of its constituent elements’ properties and powers. A relational structure combines basic elements—such as the subjects of a social relation—in a new way. These elements cannot be explained based on their former properties and powers because they belong to a new relation. For example, in an interview situation, two or more people form a relationship that creates a new kind of situation. In this new situation, individuals use their properties and resources in a novel way as the situation develops relationally. These individuals receive positions of power through this new relational situation, which they apply during the situation (Raittila & Vuorisalo, 2021). Thus, individuals give rise to social forms that do not depend on the individuals themselves but are, rather, products of the individuals’ actions relative to other individuals in a given context (Donati, 2015, p. 88).

In the following empirical analysis, we utilize the concept of *space*, and particularly *socially produced*, *relational space*. In line with relational ontology, *space* is understood as a continuously negotiated, reconstructed, and reorganized phenomenon (Fuller & Löw, 2017; Lefebvre, 1991; Löw, 2016; Massey, 2005; Soja, 1996). We approach *research space* as the ongoing production of a *relational space* where the physical environment, personal interpretations of physical and cultural spaces, and culturally and collectively shared views of space entwine (Soja, 1996, p. 62–70; Raittila, 2008, p. 26–29; Vuorisalo, Rutanen & Raittila, 2015)*.* Thus, we understand a *research space* to comprise the diverse aspects of the research process. *Research space* is not only constructed socially during data collection but also extends beyond actual face-to-face encounters among researchers and participants. Following the *relational space* concept, the construction of a particular research space begins before actual meetings, during diverse planning for and discussions about the research process. Ideals, expectations, and goals are discussed and written, alongside particular theoretical, methodological, financial, legislative, and ethical constrains that canalize the space’s construction. Additionally, even if researchers are not present daily at an ECEC center, their research space exists there; parents and teachers become aware that they are participating in a study that harbors particular interests, which might lead to changes in practices beyond the study’s data collection situations. Finally, all participants personally interpret what transpires during data collection situations, as well as the focus of a research project. Thus, *research space* is the social and spatial context for ethics-in-action.

## Data for This Article

This article is based on a project that has focused on children’s transitions to and within ECEC settings (Trace in ECEC - project, including collaboration in ISSEET - International Study of Social Emotional Early Transitions - project, White et al., 2020).^2^*Educational transition* refers to a person’s move from one institutional setting or phase to another within the educational continuum formed by diverse educational institutions (Perry et al., 2014; Vogler et al., 2008). These transitions often occur between spatially segregated, age-specific groups in educational institutions, and they effect a change in cultural settings and their embedded values, routines, practices, and rules (Dunlop & Fabian, 2006). The Trace in ECEC - project aimed to identify and investigate the main educational transitions for children in Finnish ECEC. We predicted at least the following major transitions: the child’s first entrance from home care into ECEC, and then the child’s transition into a new age-based group or setting until their pre-primary education at six years of age.

In this article, we focus on the specific research relationship with one child, whom we have observed since 2016. At the time of writing this article, our study has lasted five years. This child started ECEC when she was 18 months old. During the years of our ongoing research project, instead of undergoing only one transition for the group of children over 3 years old, the child moved from one age-based group (and “room”) to another annually. When the child started attending her ECEC center, the project’s data collection included observations and video recordings in a 7-month period. During the child’s later transitions, one to two days were observed and video-recorded. The project’s data collection also included semi-structured interviews with parents and teachers. Updating consent forms and privacy notices was also necessary, almost annually. All of these regularly occurring meetings, observations, and discussions challenged us to reflect on relational ethics.

## Methods and Analytical Approach

In this article, we focus on *critical incidents* (Halquist & Musanti, 2010; Tripp, 1993) during different moments of our study’s data collection process with our project’s focus child. “Critical incidents” can be defined in a variety of ways (see Halquist & Musanti, 2010). They often point to an event or situation that stands out, causing reflection and development. Following Halquist and Musanti (2010), we apply Tripp’s (1993, p. 8) notion that “critical incidents are not ‘things’ that exist independently of an observer and are awaiting discovery like gold nuggets or desert islands, but like all data, critical incidents are created. Incidents happen, but critical incidents are produced by the way we look at a situation.” Thus, researchers’ interpretation of an event’s significance makes an event critical, and criticality “provokes the need for reflection on that particular event, thereby rendering it visible and susceptible to further analysis and interpretation” (Halquist & Musanti, 2010, p. 450).

The analysis for this article extends in time and space. The first notes and reflections were done during the visits and data collection days in the center (2016–2021). While we were observing at the center and doing the video recordings and interviews, we also noted down theoretical and methodological reflections and discussed those regularly within the team. Ethical questions were often raised in those discussions. Some reflections linked to ethics in video methodologies and international collaboration are already reported elsewhere (Rutanen et al., 2018; see also White et al. 2020). For this article, inspired by the discussions in “Arena Ethics” (Hilppö et al., 2019), we returned to these experiences, reflections, and interpretations raised and written down during the years and observed them again, with an intention to identify from the field notes and reflections particular turning points that changed something in our approach. Thus, as a selection criterion for this study, we chose to focus on the events and incidents that “surprised the researcher” (see Halquist & Musanti, 2010) and, because they were “unpredictable” and “messy” (Tutenel et al., 2019), caused some ethical tension that led the whole research team to reflect on ethics-in-action in our situation with the study’s focus child. A common feature of these events and incidents was that they involved a challenge or contradiction to a planned or predicted occurrence during data collection.

It is clear, however, that the research process involved very diverse questions and challenges, ranging from aspects linked to formal procedures (e.g., misunderstood consent forms, challenges in communicating with families from diverse cultural backgrounds) to events in the center (e.g., people from other groups entering to the video scene by surprise). For the purpose of this article, however, we focused on the critical incidents that included the focus child’s contributions to restructuring the research space. The common nominator was that a particular, more narrow, position was assumed and expected for the child, and this position was challenged in the flow of events causing ethical reflection already while being in the center. Now, returning to the notes and written discussions, these events and interpretations were observed and reflected in the context of the whole research process, and theoretically, in terms of child’s contributions in restructuring the research space. We were able to interpret that the child’s contributions in restructuring the research extended beyond these face-to-face events and caused further consequences for the study’s research process. Thus, we identified these events and incidents as certain “turning points” that occurred (a) during the first year of our data collection in 2016, (b) in the middle of our data collection process, and (c) after four years of data collection, in 2020. The final of these turning points occurred during the COVID-19 (coronavirus disease 2019) pandemic.

## Results: the Focus Child as an Active Participant in Negotiating Ethics-in-Action

In this section of our article, we present the ways in which ethics-in-action is negotiated during the construction of a research space. We focus on a child as a research project participant as she brings resources into relational situations, actively using these resources to shape situations. However, as per our relational premise, the child alone does not build a situation or influence a situation’s construction; rather, her initiatives intertwine with the actions and interpretations of other parties involved in a research project. Thus, relations and ethics-in-action are present as processes in situations.

We identify ethics-in-action through three types of critical incidents: (a) a focus child’s spontaneous contributions to interviews, (b) interdependencies in the relationships between a child and diverse researchers, and (c) a child’s evolving expertise about data collection, which restructures the research space. During the analysis of our project’s research data, we noticed these themes in the construction of our research space and its related ethics-in-action. Here, we present these themes and reflect upon them in detail through selected critical incidents.

### The Focus Child’s Spontaneous Contributions to Interviews

During the first year of our study’s data collection process, one critical incident related to the negotiation of ethics-in-action was linked *to the focus child’s spontaneous contributions to interviews.* As a starting point, our study focused on the child’s process of transition, and the child’s transitional experiences were an interest. Regardless of this explicit starting point, the study’s research space initially emphasized adults’ constructions through spoken and recorded observations. For example, during the project’s interviews—particularly at the beginning of the process, when the focus child was about one year old—the child’s teachers and mother were originally offered positions as informants about the child’s transition. Experiences that the child had lived through were interpreted and discussed among adults. Thus, even when the child was present, power hierarchies in the research space afforded more room for adult interpretations of the child’s process than the child’s own contributions.

However, through a more detailed analysis of the project’s second interview, we observed that even during interviews with adults, the child actively contributed to interpretations and constructions of the research space. Löw (2008) uses the term *spacing* and refers to moments when people are positioned or self-positioning in relation to other people in a relational space. In this critical incident, the child’s position as a research informant and a reflector of her own experiences changed from her original position as the child actively repositioned herself. The child’s mother and the researcher accepted the child’s initiatives and, thus, reinforced an ethical stance that granted the child a strong position in the context of reviewing the research data. The following vignette (Vignette 1) from an interview also illustrates how fragile and easily hidden child’s contributions—mainly nonverbal communication—can be. This vignette also underlines the ethical commitment to creating space for the child to express these contributions.

#### Vignette 1: The Focus Child’s Spontaneous Contributions

The child was 22 months old, and the researcher (the first author) was at the child’s home, conducting the interview. The child’s mother had arranged some toys as entertainment for the child, who was on the floor. The researcher and the child’s mother were seated close to the child, on small-sized children's chairs. The conversation flowed mostly between the child’s mother and the researcher, but the child was also addressed periodically (e.g., “Isn’t that right, (child’s name)?”), and some conversation addressed the child’s playing and actions during the interview.

At one point during the interview, the researcher showed videoclips from the center to the child’s mother and invited her to comment and “think out loud” about what she saw. The laptop playing the clips was placed on a low table.

Researcher: Well, I will go back a bit [on the video] as [name of the child] was there. Then you were upset a bit [addressing the child, as the child was close to the laptop, watching the video].

Mother: I will move this a bit. [Moves the screen to see the clips better.]

Researcher: Yes. Then they [staff members] went to get your trolley, and you went to the museum.

The child: “Cry” (ikkee in Finnish, a vocalization from the verb itkee).

Mother: Cry, yes. You cried because this was a long time ago, and you had not stayed alone in the center yet.

This vignette illustrates how the space of the “interview with the mother,” as planned and indicated originally in the parents’ consent forms, turned into a shared research space. The child’s mother and the researcher addressed the child directly since she was present (“you”), and the child recognized and related to the situations depicted in the video clips. The interview encounter unfolded in a time and space that extended back to the center and its institutional space. The laptop, the video images, the child’s close proximity to the adults, the conversation around and with the child, and the child’s ability to reach toward the laptop were all interlinked as an apparatus that enabled the child’s participation in the interview event.

This apparatus could also be understood as *spacing* (Löw, 2008) in the constitution of a relational space. Fritz and Binder (2018) define the *distance of goods/things* as one analytical indicator of relational space; however, *placing social goods/things* does not straightforwardly mean that goods/things would become part of a common space for everyone, as in our case, when the laptop was first meant only for the adults’ use. An active actor (the child) is needed to create a relation and utilize its potential affordance (Gibson, 1977)—namely, the laptop and video images—to make them part of her own relational space, too. Thus, the child entered the research space and process not only as an object of study and a focus of the adults’ discussions but also by transforming the research space relationally with her presence, gestures, initiatives, and vocalizations.

This interview event underlined the child’s position as knowing about and constructing the research space from her own embodied experiences. Among the interview participants, only the child herself was part of the studied child group, and she related to the space, as well as the children whom she saw in the video clips. Thus, the child contributed significantly to the knowledge production that took place during this interview. The interview’s relational space changed in form and shape as the child changed its relational balance and introduced new properties and powers. With words, vocalizations, pointing, and other gestures, she was able to express views and direct the adults’ attention to herself and *her own experiences* in relation to the content in the videos. The interview participants’ positions changed. Eventually, instead of talking *about* the child, the adults who were present talked *with* the child, and relations were combined in a new way.

Following the formal ethics procedures and descriptions laid out in the study’s original consent forms, many aspects of the pre-planned and structured interview situations supported the recording of adults’ knowledge and, initially, the focus child’s mother served as a translator or mediator for the researcher about the child’s gestures and vocalizations. However, we came to regard the incident described in Vignette 1 as one critical incident that led us to question our original idea that the child should participate in interviews only later in the data collection process, once she had reached the age of three, as the project’s original plan and parent consent forms described. The incident in Vignette 1 clearly showed that even the very young child’s embodied expertise about events at the ECEC center demanded our research attention, given our ethical commitment to focus on the child’s views of the studied transitions.

### Interdependencies Between the Focus Child and the Study’s Diverse Researchers

Another critical incident occurred three years after the start of the project. This incident was linked *to the evolving and mutually constituted personal relationship between us researchers and the focus child during the years of the study’s data collection process*. Since the study continued for many years, we encountered numerous opportunities—particularly for the first author—to interact and discuss diverse issues with the child. By this critical incident, the focus child had become aware of her particular position as a research subject. This position had become clear to her not only through her discussion with her mother and the first author but also during repeated encounters in which the first author had directed a video camera toward the child. She had been the focus of the first author’s attention, and she had enjoyed having repeated joint conversations with this researcher, whom she was already familiar with, and seeing herself in videos during the interviews with her mother.

The study’s free-flowing discussions and data collection days, which involved structured observations and video recordings, created some ethics-in-action tensions. From the position of a researcher, the first author was able to distinguish moments when she played the formal role of researcher as an observer and as a video recorder. As the study’s consent forms for ECEC teachers expressed, these two roles and activities intended to disturb the “everyday life in ECEC” as little as possible. However, during the flow of our data-collection interactions with the child, we came to realize that we had indeed changed the “everyday life” at the center. As Hilppö et al. (2019, p. 406) write, “There is no neutral position, no place from where we can look at the world as if detached from its events, as if not responsible for what is going on.”

From the events at the ECEC center, we were able to interpret a particular “critical incident” on one observation day that left us, as a team, reflecting upon our project’s ethics-in-action and boundaries. On that day, two researchers were performing the observational role—particularly, the first author (who already had a 3-year relationship with the focus child at this point) and the third author (who was newly acquainted with the child and the ECEC center). An aspect of the exchanges that occurred between the researchers and the child that day was challenging to interpret—so much so that these exchanges made the whole team to reflect critically on the nature and expression of the child’s assent to participate in the study. While we (author 1 and 3) were at the ECEC center, the child could not physically escape the research situation, but other signs had shown that she objected to something about either the situations or the exchanges themselves, and she played an active role in restructuring interactions and the research space in directions that we had not planned.

The following vignette (Vignette 2) illustrates the incidents that day that challenged our whole teams’ expectations of how the project’s data collection would and could occur. We realized that simply replacing one researcher (the first author) with another research (the third author) in the observing and video-recording roles was not possible because the two researchers’ relationships and previous histories with the child differed significantly. While the child was accustomed to encountering the first author sometimes as the “silent” video recorder and sometimes as an active discussion partner, the child’s actions toward the third author called for this newly introduced researcher’s active engagement; the child did not accept the third author’s position as a silent observer.

#### Vignette 2: Interdependencies and a Call for Active Engagement

This incident occurred on a day when the focus child had returned to the ECEC center after a holiday. Two researchers were present—one of whom (the first author) was familiar to the child and one of whom (the third author) was not. The third author, unfamiliar to the child, was video-recording the child’s arrival at the center.

The child asked her mother, “Why she doesn’t speak?” and the child’s mother answered, “Because they are researching and recording the day.” The third author presented herself (in a low voice) to the child and said that she was doing the recording. The third author followed the child through a corridor to the child’s locker, where she was about to leave her outdoor clothes. No one else was present in the corridor. The child gazed and frowned at the camera and the third author, who was behind the camera. The child turned her back to the third author.

Later that day, the child posed questions to both of the researchers who were present—the first and third authors. The child was sometimes physically very near while talking, for example, when putting a hat on the third author or opening the third author’s coat zipper. The child also playfully approached and teased both of the present researchers, first talking to them and then hiding behind a bench; “You can’t get me at all to the tapes!” (Et yhtään saa mua nauhalle!) she said. The child’s teachers also commented that the child had had her “own show” during naptime because of the unfamiliar third author observing the child: she was acting like a ghost and moving around more than usual. Later that day, the child was throwing sand toward both of the researchers during outdoor time, while they were writing notes.

Vignette 2 describes how the child had to face a confirmation of the research space’s structure (first constructed a long time ago) by the adults that somewhat conflicted with the interactional structures that had developed outside the official research activities. Löw (2008) defines *rules embedded in institutions* as one structural element of *relational space*. The institutional research rules (i.e., informed consent) agreed upon by the adult parties in the research had defined the child’s day and the relational spaces she was involved in. Her relational space was invaded first by silent video recording, as the child’s mother confirmed. Without any possibility of repositioning herself, the child remained first an object of filming. Our (author 1 and 3) active engagement in pre-structured data collection (silent observation, focused on video recording and writing notes) obviously constrained interactions, distinguishing them from discussions with the child.

Throughout the day when Vignette 2 occurred, however, the child challenged our (authors 1 and 3) role as observers, as well as the silence and objectification linked to this role. The child approached both of the present researchers to show and discuss various issues, and long conversations took place with the child, but the engagement between the child and the researchers was also nonverbal—including her throwing sand toward us. All the physical elements constituting the relational space were embedded indoors and outdoors, such as social good, institutional rules, and the placing of people (Löw, 2008). Fritz and Binder (2018) also noted that actors and their distance and closeness in a relational space are the analytical elements that also arise in participation spaces. Since the researchers were present observing the child, her teachers sometimes withdrew from the observation situations. This withdrawal was also significant to the event’s unfolding—for example, the teachers were not present to intervene even when the child threw sand outdoors.

These incidents in which the child objected something also demonstrate the power inequality linked with children’s difficulty of withdrawing their participation from research (Einarsdóttir, 2007) and the challenging question of what children’s withdrawal looks like. Did the child’s frowning described in Vignette 2 indicate disapproval, signaling a lack of assent to continue recording? Or, given the silence in the situation, did the child frown at the silence, hoping the third author would talk to her like the other newly introduced adults who had entered the ECEC center had spoken with the child? Was throwing sand toward the first author, with whom the child had a long-term relationship, an invitation to play and engage—or an objection to being recorded and observed? Obviously, the research space included the child’s diverse initiatives and even forbidden actions. These elements prompted the team to consider whether the child had tried to reformulate her position in relation to us by breaking rules. For instance, the sand throwing—an example of a forbidden action—could be interpreted as an attempt to reorganize and challenge the research space that included us (authors 1 and 3) as silent observers who did not actively, verbally engage with the child. This day’s critical incident also clearly indicated the diverse histories and interdependencies between the child and different researchers, serving as a reminder that these interdependencies should be carefully acknowledged—not ignored—in data collection and ethical decision-making (see also Hilppö et al., 2019).

### The Focus Child’s Evolving Expertise in Data Collection and Its Restructuring of the Research Space

The third critical incident occurred four years after the start of the project’s data collection, and it is linked to the focus child’s active position in restructuring the research space through her *evolving expertise in data collection.* This incident serves as an example of the research process’s unpredictability and the related ethics-in-action associated with long-term relationships. Additionally, this critical incident illustrates the clear tension between the formal aspects that can be agreed upon with parents in advance and such formal consents’ inability to address actual encounters’ ethics-in-action, particularly in terms of the focus child’s contributions to the research space, which were impossible to predict in full detail. The question of children’s assent is also central to this incident, prompting the questions of what a young child’s assent to a study looks like in practice and how to evaluate the boundaries of a child’s assent.

This critical incident occurred in 2020, when the interviews were framed differently because of the COVID-19 restrictions imposed in 2020. These interviews—which covered COVID-19–related transitions in spring 2020, and then the child’s transition to a new center after the summer—took place online, with both the child’s mother and the child present. The child’s mother and the third author, who were involved in these online interviews, had mutually agreed that the child could, and even should, be present. By now, over the years of the research, the child’s mother had already explained to the child various times that the researchers were interested in her experiences of transitions. The child’s mother had framed the child’s opportunity to participate in our study as positive—as a privileged position to hear and learn from the child’s everyday life, particularly through videos. The following vignette (Vignette 3) illustrates the unpredictability of and challenge to remaining open to the child’s suggestions. It also illustrates how the child’s lived expertise had evolved in relation to the research space’s meaning—and what happens vis-à-vis data collection in a socially constructed research space, breaking and challenging that structure.

#### Vignette 3: The Focus Child’s Expertise in Data Collection

This critical incident occurred during an online interview through a Zoom conference call. The third author said that she had heard that the child had transitioned to a new ECEC center. The child commented “yes” right away but went on to say that she didn’t want to tell and that no one should say. The mother asked the child why.

The child answered: I want to keep it a secret. I mean you [referring to the third author] just have to guess the name!

Researcher: To guess from all the centers of the world?

Child: No, just from [name of the involved city] centers!

Both the child and the researcher sounded playful, and the researcher started to guess different names. She knew the right name, but she started with the names she knew were incorrect. The child responded “no” until hearing the correct center name.

Vignette 3 illustrates an exchange in which the child took the lead and invited the third author, to the researcher’s surprise, to engage playfully with the child in a guessing game. Our interpretation suggests that the child was aware of the researcher’s interest in certain information, and the child’s mother also underscored this position. Thus, information constituted a type of power in defining how the situation evolved and how the research space was constructed. Löw (2008) considers knowledge one of the key elements in building a relational space. The child seemed to take advantage of the information and knowledge she had gained and absorbed during previous interviews and the research process in general, a process that was also linked to the child’s having become familiar and formed a more personal relationship with the third author. The child captured the research space by positioning herself as an inquiring researcher, refusing to respond and asking her own question. She knew how an interview takes shape, through questions and answers. Thus, the child conducted the situation, playfully breaking from the traditional question-and-answer interview pattern. She led the situation differently than the previous interviews that she had been present in while still engaging in a conversation that eventually led to her revealing the new ECEC center’s name.

The interview setting was somewhat new to everyone involved since it was the second online interview with the child also present, sitting next to her mother on a couch. In the event’s material-spatial organization, the researcher was not next to the child and her mother, but both the child’s mother and the child were facing the researcher through their screen. This kind of spacing (Löw, 2008) of a research space gave the child an opportunity to move around the room—sometimes approaching the screen and showing some toys or other objects from her home, and sometimes distancing herself, withdrawing from the screen and the conversations, and also playfully “hiding” from the screen. This spatial arrangement—that is, these processes of placing people and goods (Löw, 2008)—enabled the child to better regulate her participation and position in relation to the event, either being visible or hiding, but at the same time listening carefully to what was being said about her experiences at the center. Regarding ethics-in-action, this critical incident underlined that even within a pre-planned interview structure, actual interview encounters are more organic and fluid, such that assent to engage in conversation is negotiated and interpreted throughout an interview. Additionally, an interview’s actual content ultimately takes shape during the interview itself, based on the relationships and previous histories between the situation’s participants. In Vignette 3, the young child had gained considerable information and insights about the interests and processes surrounding the research project, which led her to steer the situation in directions that she felt comfortable with and interested in.

## Discussion and Concluding Remarks

### Research Space as an Intervention

Inviting an ECEC center and a child’s parents to participate in a study directs the attention of everyone involved in such a project to the study’s focus—in our case, children’s transitions in ECEC. This paper’s example of a long-term research relationship with our child subject and her family enabled an exploration and interpretation of some of the ways in which research occurs as an intervention. Participation in research, especially ethnographic research, changes the everyday lives of everyone involved. By applying a relational approach and spatial thinking, we have approached the research process as a research space that is co-created in a relational process (Lefebvre, 1991; Löw, 2008; Soja, 1996). Thus, we were able to show how ethical questions and decisions are also spatially situated in the ongoing construction of a research space. Additionally, the focus child’s position as one potential modifier of the situation highlights ethics-in-action.

The critical incidents we have discussed in this paper can be regarded as a context about which participants shared some understanding while harboring different positions. Researchers know and think differently about research situations than participating child’s parents, and all adults approach research situations differently than children. The context called “research” invites and creates a specific mental image among all participants about what will happen. However, we have shown how research spaces and relational ethics as ethics-in-action can be shaped by any participant in any position.

### Ethics-in-Action and Learning with the Focus Child

At the beginning of this article, in Introduction, we referred to the legislative framing of research projects, mentioning changes to personal data protection as one aspect (General Data Protection Regulation [EU] 2016/679). This network of legislation, ethical agreements, and procedures that are supported and required by universities and other research organizations are important in navigating the construction of a research space and ethics-in-action with participants. However, ultimately, situational and often surprising encounters allow researchers to measure how to interpret and follow ethical commitments to subjects’ rights, as well as respect for anonymity, confidentiality, and privacy. From a research ethics perspective, this situational, social construction of a research space poses many challenges. The agreements that are supposed to be made between two or more autonomous actors in advance poorly fit the actual process of ethics-in-action that evolves unpredictably during research, as we have shown with our examples in this article. Similar examples and critiques have been provided in articles in *Human Arenas*’ special section on relational ethics for psychological research, “Arena of Ethics” (Freeman, 2019; Chimirri, 2019; Hilppö et al., 2019; O’Doherty & Burgess, 2019; Søndergaard, 2019). Our commitment to our research funders and to our original research questions as the pre-planned, imaginary, and predicted space written in plans must enter a dialog with the socially constructed space that emerges and evolves throughout the actual research process.

With research involving young children, adults (teachers, parents) often occupy positions as informants about children’s everyday experiences, and researchers easily assign this position to the adults surrounding child research subjects. Our explorations of the critical incidents discussed in this article painstakingly show the hierarchies that are linked to the construction of a research space. In some moments, we could engage in what Chimirri (2019, p. 453) calls “curious co-explorations” or “what it means to be part of this world.” In other moments, attempts to pursue data-production encounters regarding our research theme (transitions) directed our exploration process. At the same time, much of what we learned and how we learned from encounters with our project’s focus child, other children in the center, and the events we jointly lived through had not been predicted. This unpredictability led us to reflect on the concept of *transition* and how it unfolds in actual situations we jointly constructed during our research process. We also observed different interpretations and alternative, sometimes complementary, but also conflicting views among the child, the child’s mother, the child’s teachers, and ourselves as researchers about what took place and how the child had experienced transitions. These different perspectives led us to reflect on such differences as “potentiality in knowledge generating encounter” (Chimirri, 2019).

### Critical Incidents as an Analytical Tool

This study challenged us with the idea to explore and reflect on our study’s critical incidents since they pointed us to some uncomfortable, challenging moments in which we, as researchers, did not always have clear answers about how or whether to take responsibility in guiding the research process. Unexpected and unpredictable critical incidents occurred that led us to reflect on the value of indeterminacy, which is so deeply embedded in knowledge production during research (see also Chimirri, 2019). At the same time, such significant moments encouraged our reflection on ethics that are lived through during the construction of a research space as ethics-in-action. Thus, the production of a research space and the consequences of that construction very much align with Chimirri’s (2019, p. 455) elaboration, based on Bohr (c.f., Thiele, 2014; Barad, 2007) and Barad’s (2007) work that we “cannot beforehand know where our encounter will be taking us.”

Although our examination of critical incidents enabled us to focus on ethically challenging events and situations during our project’s data collection, our approach faced some limitations. The studies that Halquist and Musanti (2010) analyzed as examples in developing the critical-incident approach underlined the value of using critical incidents insofar as they allow research participants and researchers to “engage in systematic and collective inquiry of issues of power, structure and relationships” (Halquist & Musanti, 2010, p. 459). Our approach did not come close to engaging participants in these kinds of ongoing discussions and interpretations. Instead, we have taken the first steps toward interpreting critical incidents from a research team’s point of view, attempting to elucidate some of the ethical challenges that face research with young children at ECEC centers.

### Concluding Remarks

In this paper, we have explored how ethics-in-action is negotiated in the construction of a research space that involves a long-term research relationship with a young child. As our analysis of some critical incidents has illustrated, ethical questions can be unpredictable, and they cannot be separated from the mutually constituted relationships or socio-spatial context in where they emerge; thus, they are relationally and spatially embedded. These sometimes very thorny questions offer in turn valuable perspectives that would not have become part of our researcher expertise if we had not encountered conflicting situations during data collection.

Our long-lasting research relationship with our study’s focus child elicited the child’s wide range of active participation vis-à-vis ethics-in-action. This paper has shown how the young child actively participated in constructing a research space—from the project’s first observations, when she was one-year-old, to the interviews that took place four years later. Further, this paper has illustrated how diverse (playful, formal, and personal) relationships are linked to how ethics-in-action occurs during actual research encounters.

An anticipation of research-related ethical principles and issues is mandatory for researchers, and this anticipation has been extensively guided by research organizations as well as ethical boards and committees. However, in this article, we have shown that even careful preparation and planning for the research process is able to cover only some of the issues that will emerge. This partiality is linked to the profound interdependencies between the research process, relationships and persons in research space. Similar to the relationships and relations being unique and continuously evolving, so are the ethical questions linked to the research space that is being socially constructed throughout the process that may last many years.

In this article, we ended up acknowledging the unpredictably of research situations, but also offer some tools to work with ethics-in-action instead of trying to eradicate the ethical dilemmas. In our view, it is important to understand that (1) all involved in research situation are creating research space where ethics evolves, (2) even a non-verbal child can utilize the embodied participation in construction of particular research ethics, (3) every child has her/his own ways to participate in the study, (4) interdependencies between a child and various researchers should be carefully acknowledged during data collection and ethical decision-making, and (5) all participants, even children, are able to develop insights about the processes of research project through repeated, diverse encounters with researchers and research methodologies. Research practices need to be challenged to ensure that there is space for all parties involved to comment or show their views, that all those are heard and taken into account. This means that not only decisions and guidelines of the ethics committees are followed but also relational ethics are lived-through.

The contribution of this article is based also on the relational and spatial approach that we used to analyze the “critical incidents” in construction of research space. This approach enables the exploration of research process within its relationality as unique process, including diverse relationships between the participants. For us, this allowed to shed light to child’s spontaneous, nonverbal contributions that may easily remain unnoticed. In addition, the focus on research space caused reflection on child’s assent as contextually and spatially embedded and complex, continuously negotiated phenomena. Thus, research space is a joint product, where the child is an active participant with particular contributions. We are left to confirm, based on our experiences, reflections and analyses, that ‘unpredictability’ and ‘messiness’ are central aspects in research and ethics-in-action in research with children.

## Footnotes

1 Regulation (EU) 2016/679 of the European Parliament and of the Council of 27 April 2016 on the Protection of Natural Persons with Regard to the Processing of Personal Data and on the Free Movement of Such Data, and Repealing Directive 95/46/EC (General Data Protection Regulation), which became enforceable in Europe in 2018.

2 *Early childhood education setting* is a general term that can refer to diverse settings—for example, to “open daycare centers” and center-based early childhood education. To refer to center-based ECEC, the term *daycare center* (*päiväkoti*) is used in Finnish. The term is controversial since it underlines care instead of both education and care. Thus, we will use the term *ECEC center* or *center* throughout the text to refer to the centers that we studied.
